# Gaps in COPD Guidelines of Low- and Middle-Income Countries: A Systematic Scoping Review

**DOI:** 10.1016/j.chest.2020.09.260

**Published:** 2020-10-08

**Authors:** Aizhamal Tabyshova, John R. Hurst, Joan B. Soriano, William Checkley, Erick Wan-Chun Huang, Antigona C. Trofor, Oscar Flores-Flores, Patricia Alupo, Gonzalo Gianella, Tarana Ferdous, David Meharg, Jennifer Alison, Jaime Correia de Sousa, Maarten J. Postma, Niels H. Chavannes, Job F.M. van Boven

**Affiliations:** a Groningen Research Institute for Asthma and COPD (GRIAC), University of Groningen, University Medical Center Groningen, the Netherlands; b Department of Pulmonary Diseases, National Center of Cardiology and Internal Medicine, Bishkek, Kyrgyzstan; c UCL Respiratory, University College London, United Kingdom; d Hospital Universitario de la Princesa, Universidad Autónoma de Madrid, Madrid, Spain; e Division of Pulmonary and Critical Care, School of Medicine, Johns Hopkins University, Baltimore, MD; f Department of International Health, Bloomberg School of Public Health, Johns Hopkins University, Baltimore, MD; g Center for Global Non-Communicable Disease Research and Training, Johns Hopkins University, Baltimore, MD; h Woolcock Institute of Medical Research, Sydney, Australia; i South Western Sydney Clinical School, University of New South Wales, Sydney, Australia; j Division of Thoracic Medicine, Department of Internal Medicine, Shuang Ho Hospital, Taipei Medical University, Taipei, Taiwan; k University of Medicine and Pharmacy ‘Grigore T. Popa’ Iasi (UMF Iasi), Iasi, Romania; l Biomedical Research Unit, A.B. PRISMA, Lima, Peru; m Universidad de San Martin de Porres, Facultad de Medicina Humana, Centro de Investigación del Envejecimiento (CIEN), Lima, Peru; and the Universidad Cientifica del Sur, Facultad de Ciencias de la Salud, Lima, Peru; n Department of Medicine, Makerere Lung Institute, Kampala, Uganda; o Department of Medicine, School of Medicine, Universidad Peruana Cayetano Heredia, Lima, Peru; p ARK Foundation, Dhaka, Bangladesh; q University of Sydney, Faculty of Medicine and Health, Australia; r Life and Health Sciences Research Institute (ICVS), School of Medicine, University of Minho, Braga Portugal; ICVS/3B’s, PT Government Associate Laboratory, Braga/Guimarães, Portugal; s University of Groningen, University Medical Center Groningen, Department of Health Sciences, Unit of Global Health, Netherlands; t Department of Public Health and Primary Care, Leiden University Medical Center, Leiden, the Netherlands

**Keywords:** chronic obstructive, consensus, developing countries, pulmonary disease, reference standards, GACD, Global Alliance for Chronic Diseases, GNI, gross national income, GOLD, Global Initiative for Chronic Obstructive Lung Disease, HIC, high-income countries, IOM, Institute of Medicine, LMIC, low- and middle-income countries, PRISMA, preferred reporting items for a systematic review and meta-analysis

## Abstract

**Background:**

Guidelines are critical for facilitating cost-effective COPD care. Development and implementation in low-and middle-income countries (LMICs) is challenging. To guide future strategy, an overview of current global COPD guidelines is required.

**Research Question:**

We systematically reviewed national COPD guidelines, focusing on worldwide availability and identification of potential development, content, context, and quality gaps that may hamper effective implementation.

**Study Design and Methods:**

Scoping review of national COPD management guidelines. We assessed: (1) global guideline coverage; (2) guideline information (authors, target audience, dissemination plans); (3) content (prevention, diagnosis, treatments); (4) ethical, legal, and socio-economic aspects; and (5) compliance with the eight Institute of Medicine (IOM) guideline standards. LMICs guidelines were compared with those from high-income countries (HICs).

**Results:**

Of the 61 national COPD guidelines identified, 30 were from LMICs. Guidelines did not cover 1.93 billion (30.2%) people living in LMICs, whereas only 0.02 billion (1.9%) in HICs were without national guidelines. Compared with HICs, LMIC guidelines targeted fewer health-care professional groups and less often addressed case finding and co-morbidities. More than 90% of all guidelines included smoking cessation advice. Air pollution reduction strategies were less frequently mentioned in both LMICs (47%) and HICs (42%). LMIC guidelines fulfilled on average 3.37 (42%) of IOM standards, compared with 5.29 (66%) in HICs (*P* < .05). LMICs scored significantly lower compared with HICs regarding conflicts of interest management, updates, articulation of recommendations, and funding transparency (all, *P* < .05).

**Interpretation:**

Several development, content, context, and quality gaps exist in COPD guidelines from LMICs that may hamper effective implementation. Overall, COPD guidelines in LMICs should be more widely available and should be transparently developed and updated. Guidelines may be further enhanced by better inclusion of local risk factors, case findings, and co-morbidity management, preferably tailored to available financial and staff resources.

FOR EDITORIAL COMMENT, SEE PAGE 465

According to the Global Burden of Disease study, more than 90% of COPD deaths occur in low- and middle-income countries (LMICs).^1^ Notably, these deaths are accompanied by a significant socioeconomic burden for patients, their families, and societies.^2^ As such, to achieve the greatest impact in reducing premature COPD deaths around the world, efforts should focus on optimizing COPD treatment in LMICs. One of the ways in which treatment can be optimized is through effective dissemination and implementation of guidelines. Guidelines should provide standardized, evidence-based prevention, diagnosis, and management recommendations. Some countries have developed their own guidelines, and from 2001 the Global Initiative for Chronic Obstructive Lung Disease (GOLD) Strategy Report has been established. Since then, multiple countries have adopted the GOLD updates that followed.^3^

Although guidelines are critical to help improving COPD care around the world,^4^, ^5^, ^6^ guidelines are of no use when poorly implemented. Notably, for proper implementation, several prerequisites need to be considered. Interventions can only be successful when affordable, practicable, (cost-)effective, acceptable, safe, and equitable (resulting in the acronym APEASE).^7^ The ability to meet these criteria differs between (and within) countries. Interventions can be feasible in one setting but not in another, depending on factors such as demographics, infrastructure, health-care budgets, culture, and environment.^8^, ^9^, ^10^, ^11^

Historically, COPD guideline reviews dating back over a decade included 15 to 41 guidelines and focused on assessing the quality of the development, content,^12^ and specific monitoring of recommendations,^13^ with limited focus on LMICs.^14^^,^^15^ The two latest reviews of COPD guidelines assessed diagnosis and treatment criteria,^16^ development, authors, and audience,^17^ but focused on European guidelines only.

We have not identified any previous review that focused on COPD guidelines in LMICs. To stimulate effective implementation of COPD guidelines in LMICs, a systematic assessment of which aspects related to development, content, or quality should be targeted is critical.

We systematically reviewed national COPD guidelines, focusing on global existence as well as on potential gaps in development, content, context, and quality that may hamper their implementation in LMICs. We therefore undertook a scoping review to identify the gaps or topics that should be prioritized in future focused systematic reviews.

## Methods

### Study Design

This global COPD guideline scoping review was informed by a systematic literature search, performed and reported (e-Table 1) according to preferred reporting items for a systematic review and meta-analysis (PRISMA) scoping review standards.^18^ Because this work did not involve human subject research, no ethical approval was required. The work is part of the Global Alliance for Chronic Diseases (GACD) COUNCIL project.

### Data Sources, Search, and Inclusion

To identify as many guidelines as possible, a sequential approach was taken. First, PubMed (February 15, 2019) and EMBASE (February 18, 2019) were searched to identify published COPD guidelines. Before manuscript submission (January 14, 2020), the search was repeated, but no additional guidelines were identified. References of previous reviews and identified articles were inspected to identify further guidelines. Additionally, authors and the GACD Research Network collaborators (all health professionals or clinical researchers specializing in lung disease) were asked to identify guidelines in their own country or guidelines they were aware of from other countries, not yet identified through the database or online searches. Also, the guideline databases of the International Primary Care Respiratory Group, Guidelines International Network, and the Tripdatabase were checked. Thereafter, authors were asked to reach out to colleagues and national (guideline) websites from all of the remaining countries for which no guideline had been identified. Similar search strategies have been used by earlier GACD guideline comparisons.^13^

The online search strategy in PubMed and EMBASE was based on the list of key words used in the previous GACD guidelines reviews,^19^, ^20^, ^21^ but with COPD as disease entity. For both databases, the search terms included *guideline* OR *consensus* OR *recommendations* OR *protocols* OR *standards* AND *COPD*. No search filters were applied, and all years and languages were considered. The full search strategy is provided in e-Table 2.

Guidelines were included as long as the following inclusion criteria were met: (1) it should focus on COPD prevention, diagnosis, and management (ie, not only focusing on specific treatments such as alpha-1-antitrypsin deficiency); (2) it should have been developed for intended use as a national COPD guideline. This could include a stand-alone document or a translation or adoption of an international document (eg, GOLD). When multiple guidelines were identified within the same country, the guideline with the largest coverage or the most recent update (ie, this could be a newly developed or updated guideline version) was selected.

### Data Extraction and Quality Assessment

Data extraction was performed using a pre-piloted digital form. A first version, based on earlier GACD guidelines assessments, was made by J. F. M. v. B. and A. T. and commented on by all authors, a combination of native English and non-English speakers with good understanding of COPD. Subsequently, small optimizations were made in an iterative process until a final version with uniform interpretation was agreed on. The final extraction form was circulated to all authors, using Research Electronic Data Capture (REDCap). Data extraction was performed by the individual authors and double-checked by a second person. For countries or languages in which authors did not have expertise, additional local clinical experts from the particular country were invited to complete and double-check the data extraction.

Data items extracted included (1) general guideline information (name, authors, year, target audience, dissemination plans); (2) coverage of specific COPD prevention, diagnosis, and management recommendations (local epidemiology, case-finding, smoking cessation, air pollution, vaccination, exacerbations, comorbidity, diet, physical activity, pharmacologic recommendations, patient education, alternative medicine, and vulnerable populations, such as indigenous people); (3) recommendations that addressed ethical aspects (eg, regarding experimental high-risk treatments, or non-evidence-based treatments), legal aspects (eg, related to end-of-life care, such as euthanasia, palliative sedation), social aspects (eg, addressing the role of informal caregivers, family, and patient organizations), and economic aspects (eg, costs, cost-effectiveness, or reimbursement); and (4) compliance with the eight Institute of Medicine (IOM) standards for optimal development of clinical practice guidelines, consisting of several evidence quality indicators (transparency of funding, multidisciplinary author composition, conflicts of interest policy, use of systematic reviews, grading of evidence, articulation of recommendations, external review, frequency of updates).^22^ The assessment of IOM criteria is further specified in e-Table 3 and was primarily chosen (rather than the more commonly used, but contentwise largely overlapping Appraisal of Guidelines for Research & Evaluation [AGREE] II tool) to be able to compare our results with previous GACD LMIC guidelines reviews that also used the IOM criteria.^19^, ^20^, ^21^ Note that the IOM standards were published in 2011. Since 2016, the IOM is known as the “Health and Medicine Division” of the National Academies of Sciences, Engineering, and Medicine of the United States.

### Outcomes by Income Status

Outcomes included global guideline coverage, defined as the absolute number and percentage of people in high-income countries (HICs) and LMICs covered by a national COPD guideline as part of the total HICs’ and LMICs’ population. Additionally, the four themes as specified under data items (general guideline information, guideline content, ethical, legal, socio-economic aspects, and compliance with the IOM guideline standards defined as the mean number of standards fulfilled) were assessed.

Countries with guidelines available were grouped and compared by income status (as of June 2018). In particular, all identified countries were classified according to the World Bank Atlas method, also adopted by the Organization for Economic Cooperation and Development.^23^ The World Bank assigns a classification for all member countries (189) and all other economies with populations of more than 30,000. Economies were classified based on their 2018 gross national income (GNI) per capita in US dollars. World Bank classifications include low-income countries ($995 or less), LMICs ($996-3,895), upper-middle income countries ($3,896-12,055), and HICs ($12,056 or more). Thus, for our comparison, countries with GNIs per capita up to $12,055 were considered LMICs and countries with a GNI per capita of more than $12,056 were considered HICs. In 2018, this classified 218 countries in the world (total population size: 7,594,270,356) in 81 HICs (37%) with a total population size of 1,210,312,147 people and 137 (63%) LMICs with a total population size of 6,383,958,209 people.

### Data Synthesis and Analyses

All data were summarized per country in Excel 2010 (Microsoft Corp) and presented in tables and figures for visual inspection and review. Categories were presented as absolute numbers per category and as percentages, continuous variables as mean and SD. Chi-squared tests and Student *t* tests were performed (IBM SPSS Statistics 23) to assess outcomes by income classification, where guidelines from LMICs were compared with those from HICs (statistical specifications are shown in e-Table 4). A *P* value < .05 was deemed statistically significant.

## Results

After searching PubMed, 3,030 titles were obtained, but after title screening only 90 were considered potentially relevant. In EMBASE, the search strategy resulted in 9,376 titles, and after screening, 43 were considered potentially relevant. When removing duplicates and reading full texts, a total of 27 relevant COPD country guidelines were identified. Of note, two of these guidelines were written for two countries (Australia/New Zealand and Germany/Austria); these “two-country” guidelines were only counted once in comparisons. GACD collaborators suggested 13 additional COPD guidelines not found by the PubMed/EMBASE search. Finally, the targeted search and outreach by the GACD Research Network provided 21 more guidelines, resulting in a total of 61 identified COPD guidelines for 63 countries. The flow diagram of article selection is provided in e-Figure 1.

### Global Population Coverage

**Figure 1:**
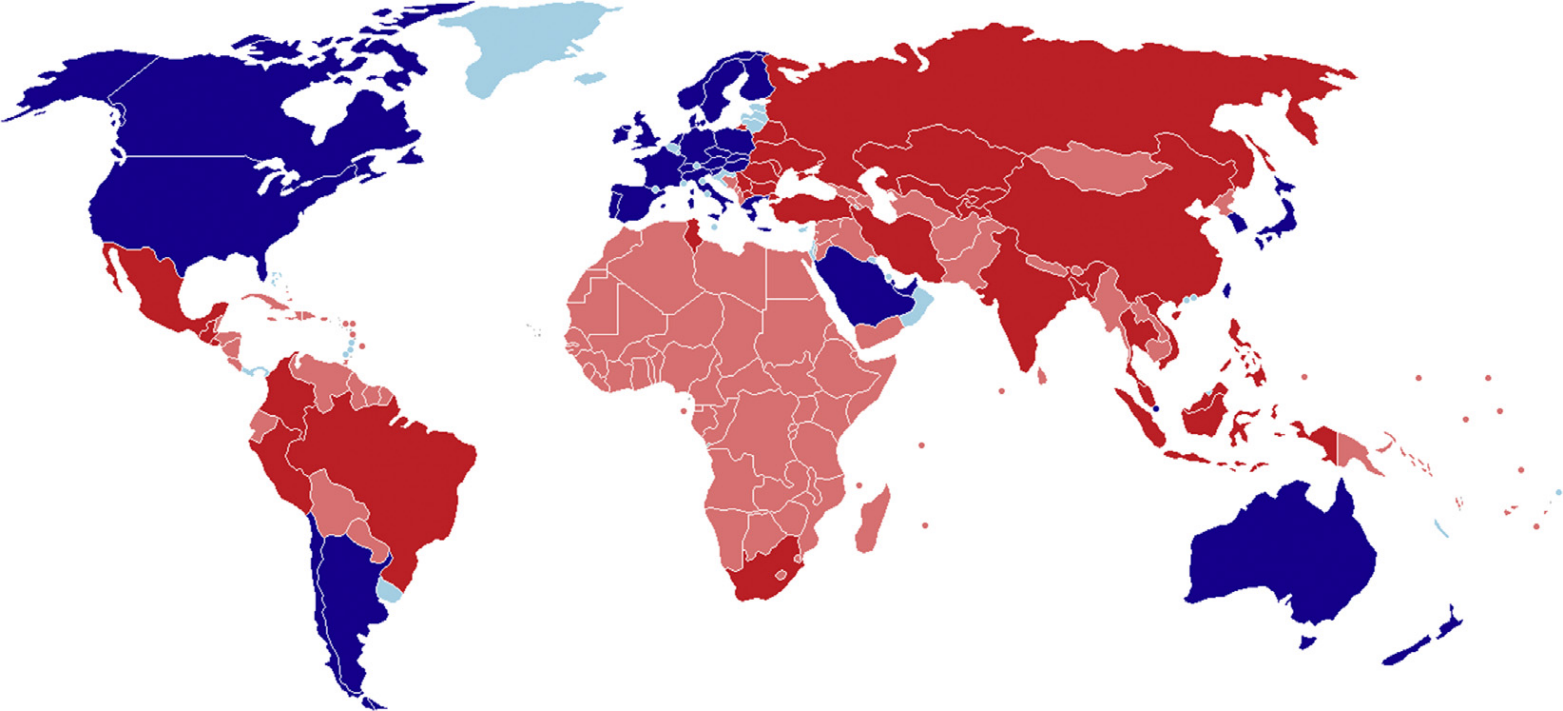
World map showing countries with and without COPD guidelines. Light blue: high-income country without country guideline; dark blue: high- income country with guideline; light red: low- and middle- income country without guideline; red: low- and middle-income country with guideline.

In total, 63 (28%) of the 218 countries with a World Bank classification had COPD guidelines (Fig 1). These 63 countries covered a total population size of 5,644,031,801 (74.3% of the world’s population). Of the 61 guidelines evaluated, 30 (49%) were from LMICs, covering 30 countries and 31 (51%) were from HICs, covering 33 countries. For LMICs, this means that 30 of the worlds’ 137 LMICs (21.9%) had their own guideline. In terms of total population, these 30 countries covered 4.46 of the 6.38 billion people living in LMICs, leaving 1.93 billion people (30.2%) without their own country guideline. In HICs, 33 of the worlds’ 81 HICs (40.7%) had their own guideline. In terms of total population, these 33 countries covered 1.19 of the 1.21 billion people living in HICs, leaving only 0.02 billion people (1.9%) living in HICs without their own country guideline. Country-specific population and income data are provided in e-Table 5.

### General Characteristics and Target Audience

Characteristics and content of all COPD guidelines are provided in e-Tables 6-18. Guidelines were mostly written by a national (respiratory) association or society or by the Ministry of Health (e-Tables 6-8). In LMICs, the oldest guideline was from El Salvador (2005), and the newest was from Bulgaria (2019).

**Figure 2:**
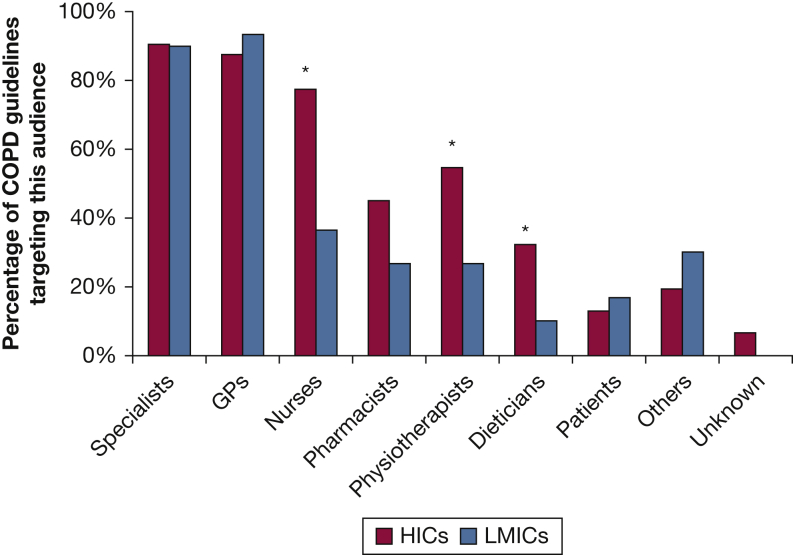
Overview of target audience of COPD guidelines around the world. ∗Significant difference, *P* < .05; GPs = general practitioners; HICs = high-income countries; LMICs = low- and middle-income countries.

An overview of the target audience of COPD guidelines is provided in Figure 2 and specified in e-Tables 4 and 9. All guidelines targeted physicians, often both respiratory specialists and primary care physicians/general practitioners, regardless of income group. LMIC guidelines tended to explicitly target a smaller group of health care professionals compared with HICs, including significantly less often nurses (37% vs 77%; *P* < .05), physiotherapists (27% vs 55%; *P* < .05) and dieticians (10% vs 32%; *P* < .05).

### Content by Income Group

**Figure 3:**
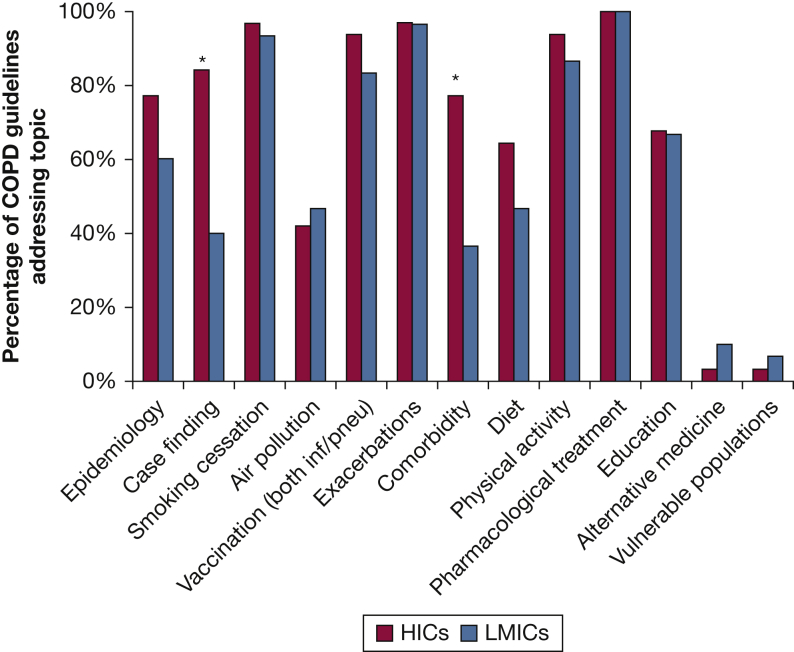
Overview of coverage of COPD management recommendations met by COPD guidelines in high-income countries and low- and middle-income countries. ∗Significant difference, *P* < .05. HICs = high-income countries; inf = influenza; LMICs = low-and middle-income countries; pneu = pneumococcal.

An overview of the content of COPD guidelines by country classification is provided in Figure 3 and specified in e-Tables 10-12. Pharmacological treatment was the only item that was included in 100% of the COPD guidelines. Compared with HICs, LMICs significantly less frequently included recommendations regarding case finding (LMICs, 40%; HICs, 84%; *P* < .05) and co-morbidity (LMICs, 37%; HICs, 77%; *P* < .05). In contrast, LMICs guidelines slightly (but not significantly) more often included alternative medicine recommendations (LMICs, 10%; HICs, 3%) and paid more attention to management of vulnerable populations (although only in two countries, Serbia and Indonesia). Of note, although more than 90% of guidelines included smoking cessation advice, management of other airborne exposures (eg, indoor and outdoor air pollution) was much less frequently mentioned in both LMICs (47%) and HICs (42%).

### Context, Dissemination, and Quality

Besides specific prevention, diagnosis, and treatment recommendations, guidelines had nonsignificant differences regarding the extent to which ethical, legal, and socio-economic context was considered. LMICs guidelines scored similarly on incorporation of ethical aspects (30% vs 29%; *P* = .93), and nonsignificantly lower on the legal (20% vs 35%; *P* = .18), social (27% vs 35%; *P* = .46), and economic aspects (27% vs 42%; *P* = .21). In LMICs, 23% of the guidelines had dissemination plans in place, whereas this was the case in 32% of HICs (e-Tables 13-15).

**Figure 4:**
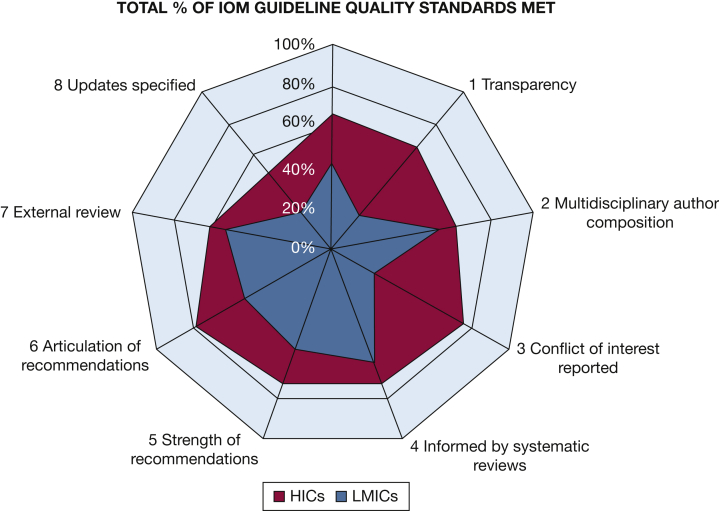
IOM guideline quality standards met by COPD guidelines in high-income countries and low- and middle-income countries. HIC = high-income countries; IOM = Institute of Medicine; LMIC = low-and middle-income countries.

In Figure 4 and e-Tables 16-18, fulfilment of IOM quality standards for good guideline development are shown for all COPD guidelines. Statistical comparisons are provided in e-Table 4. On average, LMICs guidelines fulfilled 42% (mean, 3.37; SD, 2.09), whereas HICs guidelines fulfilled 66% of the eight IOM criteria (mean, 5.29; SD, 2.02) (*P* < .05). For both LMICs and HICs, updating of guidelines was the worst scored criterion, with fewer than 50% of guidelines fulfilling this item but with better fulfilment in HICs (*P* < .05). Additionally, guidelines from LMICs scored significantly lower compared with HICs’ guidelines regarding conflicts of interests (*P* < .05), articulation of recommendations (*P* < .05), and transparency of funding (*P* < .05). If the five LMIC guidelines that were published before the IOM guidance launch (2011) were excluded, IOM criteria fulfilment was similar (mean, 3.24; SD, 2.09).

## Discussion

We assessed the availability of COPD guidelines in LMICs and identified gaps in development, quality, content, and context that may hamper their effective implementation. Regarding availability, we found a national guideline in only 30 of 137 LMICs (21.9%), whereas in HICs this was the case for 33 of the 81 HICs (40.7%). In absolute numbers, this means that 1.93 billion (30.2%) people living in LMICs were without national COPD guidelines, whereas of the 1.2 billion people living in HICs only 0.02 billion (1.9%) were without a national guideline. Regarding quality, LMIC guidelines fulfilled significantly fewer IOM standards for good clinical practice guideline development. LMIC guidelines scored significantly lower compared with HIC guidelines regarding conflicts of interests, updates, articulation of recommendations, and funding transparency. Regarding content, risk factor management was mostly restricted to smoking cessation, whereas air pollution received far less coverage despite the importance of this in causing COPD in many LMIC settings. Pharmacological treatment was covered in all guidelines, yet LMIC guidelines generally targeted a smaller group of health-care professionals, mostly physicians and significantly less often nurses, physiotherapists, and dieticians compared with HIC guidelines. Case finding and comorbidities received relatively little attention in LMIC guidelines. Regarding context, incorporation of ethical, legal, and socio-economic aspects in LMIC guidelines seemed numerically, but not statistically, lower than in HIC guidelines. Regarding dissemination, fewer than one quarter of LMIC guidelines had dissemination plans in place, compared with one third in HICs.

The difference between guideline availability in HICs vs LMICs highlights an unequivocal health disparity. Almost 2 billion people and their health-care advisors (over a quarter of the world’s population) are not directly advised on how to manage COPD according to their country context. In particular, countries in sub-Saharan Africa have no COPD guidelines, despite the existence of the GOLD Strategy Report, which can be used as a tool to frame local guidelines. The absence of national guidelines may be partly due to insufficient resources and international aid, but also could be attributable to local health, academic, and political priorities that may have historically focused more on communicable diseases such as TB, malaria, and HIV. However, given increased infection control and rising life expectancy, noncommunicable diseases such as COPD may become a new epidemic. Therefore, timely development of COPD guidelines seems key.

In some countries (eg, Romania), regional or direct GOLD translations were used. Although these translations are available, local physicians’ understanding may fall short, and more efforts on implementation of these recommendations is required.^24^ This may not only hold true for LMICs but also for smaller HICs, including Andorra, Belgium, and Luxemburg, that simply use translated GOLD or neighboring countries’ guidelines. Similarly, countries such as Uruguay and Panama mostly follow regional Latin American Thoracic Association guidelines.^25^ For LMICs this may also be the case—for example, in Middle Eastern countries that use the guideline of the Gulf Cooperation Council countries and Middle East-North Africa region.^26^

That fewer than one quarter of LMIC guidelines had dissemination plans in place underlines an important, but modifiable gap. We argue that efficient, wide-scale implementation can only be successful when effective guidelines and dissemination plans are in place, with proper understanding of local infrastructure, culture, and environment, additionally tailored to local COPD prevalence, risk factors, and resources available. Of note, most LMICs’ guidelines did not include local data and lacked economic considerations. Guidelines in LMICs tend to use some “copy paste” from HICs in relation to risk factors and did not always take into account regional differences. Notably, COPD risk factors other than smoking, such as early life disadvantages and household and ambient air pollution, are now increasingly recognized^27^, ^28^, ^29^ yet are covered in fewer than half of current LMIC guidelines. Also, regional risk differences require attention. For example, in Latin America, tobacco smoking is primarily an urban problem and is not very prevalent in rural areas. These urban-rural disparities have also been observed in Uganda.^30^ Early life disadvantages such as undernutrition also may be more prevalent in particular vulnerable populations such as indigenous and nomadic populations in both HICs (eg, Australia or Greenland) and LMICs, as well as the lack of early detection of COPD, limited access to treatment, and lack of appropriate health education and poor engagement with health resources, which is often evident in indigenous populations. Generally, research in these populations is scarce, and recommendations tailored to these subpopulations are therefore lacking.

Regarding pharmacological treatment, covered by 100% of LMICs guidelines, it is important to note that most clinical evidence to support such medicines came from trials conducted in HICs. These often included current and former heavy cigarette smokers, which do not always represent “real-world “ COPD heterogeneity^31^ and do not necessarily have the same phenotype as nonsmokers with other COPD risk factors as frequently seen in LMICs. Therefore, more trials should include LMIC populations. Furthermore, availability and affordability of recommendations should be considered, especially related to more expensive pharmacological treatment.^32^ This question was considered too detailed for this broad scoping review, and a more targeted systematic review focusing on specific pharmacologic recommendations in LMIC, considering availability and relationship with outcomes, would be highly valuable.

Regarding content, the low inclusion of case finding and comorbidity management may have to do with the relatively slower uptake of novel findings. Indeed, the GOLD report has only put more emphasis on comorbidity since 2011, and even in the current version,^3^ proper guidance on multi-morbidity is lacking. Case finding is only recommended from the 2019 update after a large HIC trial.^33^

An important finding is related to transparency of guideline development. Although proper reporting of funding and conflicts of interest are important, these are significantly less often addressed in LMICs. These issues are not restricted to guidelines and also include consideration on who funds the COPD training for clinicians. Of note, a future-focused systematic review could examine the reduced attention to conflict of interest in LMIC guidelines and whether this may have influenced the selection of medication recommendations.

Beyond COPD, similar guideline reviews comparing LMICs with HICs have been published, including those for diabetes, hypertension, and stroke.^19^, ^20^, ^21^ Although the content cannot be compared, adherence to IOM standards showed similar gaps, with a mean fulfillment of just under 2.5 IOM items for LMIC diabetes guidelines vs a mean of 5.2 in HICs. Similarly, “updating” was the lowest scored item, and the largest gaps between LMICs and HICs were related to transparency, evidence quality, and articulation of recommendations.

Given that in most LMICs no specific COPD guideline was in place, policy makers should stress the need for a COPD guideline to be developed. In doing so, there is a strong need to harmonize the methodology of guideline production and implementation. We do, however, consider that stand-alone development of guidelines with standardized methodology is challenging, expensive, and time consuming. This would include assessment of the strength of evidence with the Grading of Recommendations Assessment, Development and Evaluation approach.^34^ As such, as a minimum viable option, international guidelines (eg, European Respiratory Society, Latin American Thoracic Association) are encouraged with local context-specific adaptations beyond simple translation.^35^ Developers of future COPD guidelines should pay attention to IOM (or other recognized) standards for good clinical practice guideline development. In particular, transparency, updating, and conflict of interest management are important issues to be improved. Guideline development and targeting should include multidisciplinary experts and contain views from patients’ organizations and a public consultation process. Guidance should be provided on dissemination and implementation, including what the minimal standards of care are for each level of the health-care system.

Regarding implementation, guidelines should include what evidence the suggestions are based on. Suggestions based on studies from a country with very different context may not be implementable at all. Therefore, guidelines should include guidance on approaches to facilitate cultural adaptation and effective collaboration with vulnerable populations such as indigenous people, particularly within colonized countries.

From a research perspective, more work on barriers and facilitators to effective implementation of guidelines in LMICs is required. This would include local data collection and strategies to facilitate local adaptation and implementation of evidence, taking into account environmental, demographic, social, cultural, legal, and economic dimensions. In addition, guidelines should address the cultural needs of indigenous populations in HICs, where colonization has resulted in health inequities. Finally, when guidelines are in place, frequent updating and monitoring of adherence to specific recommendations is essential. Periodic auditing may facilitate improvement of adherence to guidelines and ultimately more cost-effective COPD care. Ultimately whether, after adjusting for income and other factors, countries with a national country-tailored guideline have better COPD health outcomes should be explored.

### Strengths and Limitations

To our knowledge, this is the first global COPD guideline scoping review and informs future guideline development around the world as well as more targeted systematic reviews. Authors and guideline reviewers represented all continents and made use of local understanding of clinical practice. Although extensive searches were performed, for some countries, general (noncommunicable disease) treatment guidelines are in place that may include treatment of various chronic diseases, including COPD.^21^ Also, guidelines that were published in English and traceable using online data sources had a higher likelihood of being included. For some guidelines, we could only find main documents, and we may have therefore missed some specific recommendations only provided in appendixes of the main document. GACD network members actively reached out to colleagues in countries for which no guidelines had been identified through online searches. Still, in some LMICs, we had no direct contacts; therefore, the establishment of a wider network of contacts is required. Having this network in place would also allow for further qualitative, in-depth data collection on physicians’ expectations and actual implementation barriers on a local practice level, and allows more targeted approaches to wide-scale guideline implementation. Given that many guidelines were only identified through the GACD network, it is not possible for independent researchers to obtain the set of guidelines used for our analysis simply by repeating the database searches with the exact search criteria. Also, we acknowledge that the AGREE II tool is currently more commonly used to assess guideline quality. However, data items largely overlap with the IOM standards.^36^ As such, we do not expect that this part of the scoping review would have resulted in different messages when the AGREE II tool would have been used instead. Regarding the content of COPD care covered by the country guidelines, we should acknowledge the review focused on broad COPD management guidelines, but that, in fact, for some aspects of COPD care, separate guidelines may be in place in some countries (eg, exacerbation management). Finally, we note that a wide range of guideline publication dates were found (2005-2019). Although we aimed to minimize potential time-related differences by performing a subanalysis for the IOM quality criteria in LMIC, this still warrants careful interpretation of the content of care comparisons.

### Interpretation

Several development, content, and quality gaps exist in COPD guidelines from LMICs that may hamper large-scale effective implementation. Of note, COPD guidelines in LMICs should be more widely available and should be transparently developed and updated. Furthermore, they may be enhanced by more focus on the inclusion of local risk factors, case finding, and comorbidity management, preferably tailored to financial and staff resources available.

## Supplementary Data

e-Online Data
