# Supplementary material for: Gaps in COPD Guidelines of Low- and Middle-Income Countries: A Systematic Scoping Review
Source: Chest. 2020 Oct 8;159(2):575–84. doi: 10.1016/j.chest.2020.09.260 (PMC7856534; doi:10.1016/j.chest.2020.09.260)
Supplement: e-Online Data [file mmc1.pdf]

CHEST Online Supplement

Gaps in COPD Guidelines of Low- and Middle-Income Countries

A Systematic Scoping Review

Aizhamal Tabyshova, MD; John R. Hurst, MD, PhD; Joan B. Soriano, MD, PhD; William Checkley, MD, PhD; Erick Wan-Chun Huang, MD; Antigona C. Trofor, MD, PhD; Oscar Flores-Flores, MD; Patricia Alupo, MD; Gonzalo Gianella, MD; Tarana Ferdous, MPH; David Meharg, MPH; Jennifer Alison, PhD; Jaime Correia de Sousa, MD, PhD; Maarten J. Postma, PhD; Niels H. Chavannes, MD, PhD; and Job F.M. van Boven, PharmD, PhD

CHEST 2021; 159(2):575-584

Online supplements are not copyedited prior to posting and the author(s) take full responsibility for the accuracy of all data.

© 2020 AMERICAN COLLEGE OF CHEST PHYSICIANS. Reproduction of this article is prohibited without written permission from the American College of Chest Physicians. See online for more details. **DOI:** 10.1016/j.chest.2020.09.260

e-Figure 1.

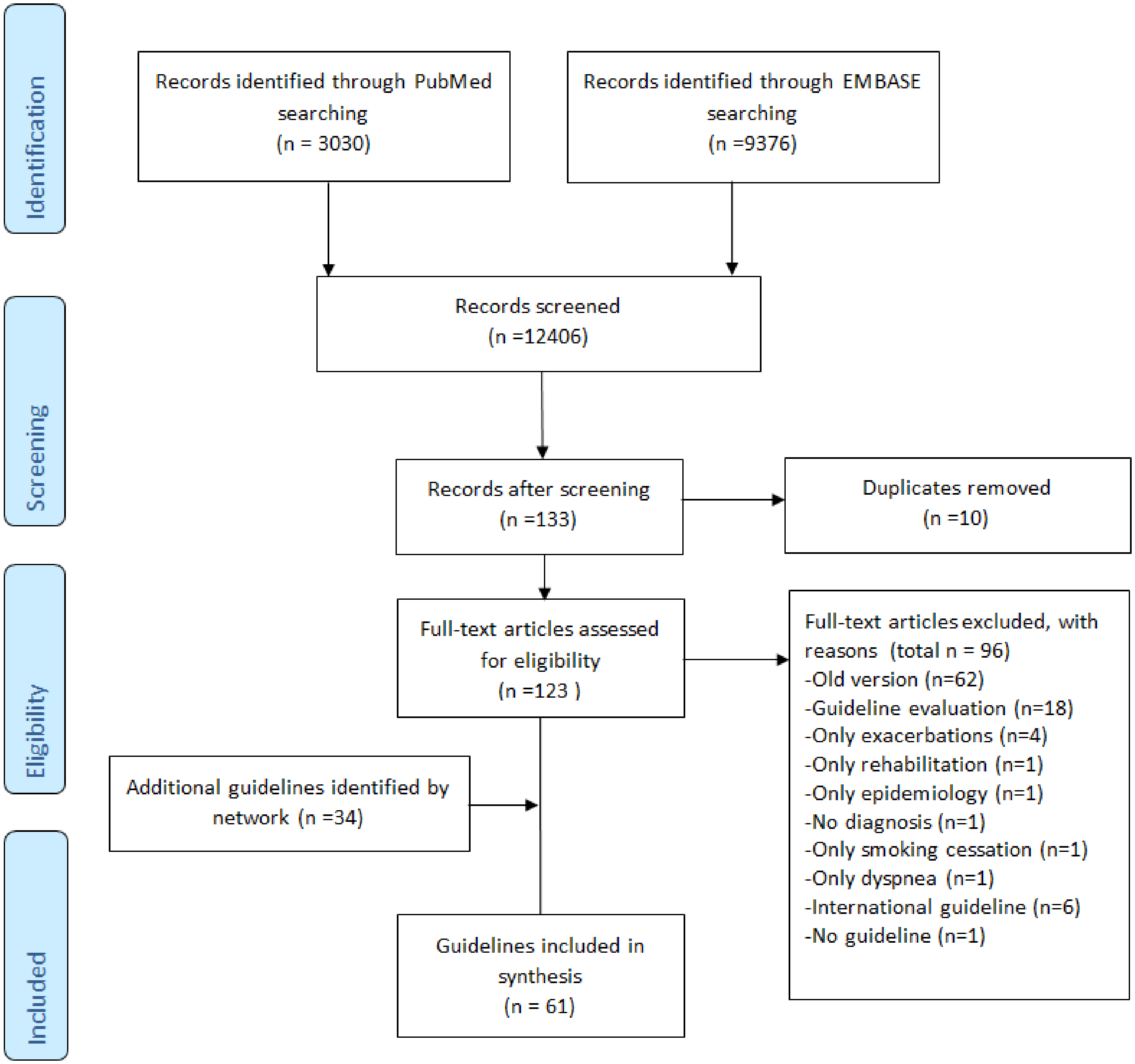

e-Figure 1: PRISMA flow diagram for article and guideline selection

Online supplements are not copyedited prior to posting and the author(s) take full responsibility for the accuracy of all data.

e-Table 1: PRISMA scoping review reporting checklist
Preferred Reporting Items for Systematic reviews and Meta-Analyses extension for Scoping Reviews (PRISMA-ScR) Checklist

SECTION	ITEM	PRISMA-ScR CHECKLIST ITEM	REPORTED ON PAGE #
**TITLE**			
Title	1	Identify the report as a scoping review.	1
**ABSTRACT**			
Structured summary	2	Provide a structured summary that includes (as applicable): background, objectives, eligibility criteria, sources of evidence, charting methods, results, and conclusions that relate to the review questions and objectives.	4
**INTRODUCTION**			
Rationale	3	Describe the rationale for the review in the context of what is already known. Explain why the review questions/objectives lend themselves to a scoping review approach.	6
Objectives	4	Provide an explicit statement of the questions and objectives being addressed with reference to their key elements (e.g., population or participants, concepts, and context) or other relevant key elements used to conceptualize the review questions and/or objectives.	7
**METHODS**			
Protocol and registration	5	Indicate whether a review protocol exists; state if and where it can be accessed (e.g., a Web address); and if available, provide registration information, including the registration number.	7
Eligibility criteria	6	Specify characteristics of the sources of evidence used as eligibility criteria (e.g., years considered, language, and publication status), and provide a rationale.	8
Information sources*	7	Describe all information sources in the search (e.g., databases with dates of coverage and contact with authors to identify additional sources), as well as the date the most recent search was executed.	7-8
Search	8	Present the full electronic search strategy for at least 1 database, including any limits used, such that it could be repeated.	8
Selection of sources of evidence†	9	State the process for selecting sources of evidence (i.e., screening and eligibility) included in the scoping review.	9
Data charting process‡	10	Describe the methods of charting data from the included sources of evidence (e.g., calibrated forms or forms that have been tested by the team before their use, and whether data charting was done independently or in duplicate) and any processes for obtaining and confirming data from investigators.	9
Data items	11	List and define all variables for which data were sought and any assumptions and simplifications made.	9
Critical appraisal of individual sources of evidence§	12	If done, provide a rationale for conducting a critical appraisal of included sources of evidence; describe the methods used and how this information was used in any data synthesis (if appropriate).	NA
Synthesis of results	13	Describe the methods of handling and summarizing the data that were charted.	10
**RESULTS**			
Selection of sources of evidence	14	Give numbers of sources of evidence screened, assessed for eligibility, and included in the review, with reasons for exclusions at each stage, ideally using a flow diagram.	11
Characteristics of sources of evidence	15	For each source of evidence, present characteristics for which data were charted and provide the citations.	Online Suppl

SECTION	ITEM	PRISMA-ScR CHECKLIST ITEM	REPORTED ON PAGE #
Critical appraisal within sources of evidence	16	If done, present data on critical appraisal of included sources of evidence (see item 12).	NA
Results of individual sources of evidence	17	For each included source of evidence, present the relevant data that were charted that relate to the review questions and objectives.	Online Suppl
Synthesis of results	18	Summarize and/or present the charting results as they relate to the review questions and objectives.	12-13
**DISCUSSION**			
Summary of evidence	19	Summarize the main results (including an overview of concepts, themes, and types of evidence available), link to the review questions and objectives, and consider the relevance to key groups.	13
Limitations	20	Discuss the limitations of the scoping review process.	18
Conclusions	21	Provide a general interpretation of the results with respect to the review questions and objectives, as well as potential implications and/or next steps.	18-19
**FUNDING**			
Funding	22	Describe sources of funding for the included sources of evidence, as well as sources of funding for the scoping review. Describe the role of the funders of the scoping review.	3

JBI = Joanna Briggs Institute; PRISMA-ScR = Preferred Reporting Items for Systematic reviews and Meta-Analyses extension for Scoping Reviews.

* Where *sources of evidence* (see second footnote) are compiled from, such as bibliographic databases, social media platforms, and Web sites.

† A more inclusive/heterogeneous term used to account for the different types of evidence or data sources (e.g., quantitative and/or qualitative research, expert opinion, and policy documents) that may be eligible in a scoping review as opposed to only studies. This is not to be confused with *information sources* (see first footnote).

‡ The frameworks by Arksey and O'Malley (6) and Levac and colleagues (7) and the JBI guidance (4, 5) refer to the process of data extraction in a scoping review as data charting.

§ The process of systematically examining research evidence to assess its validity, results, and relevance before using it to inform a decision. This term is used for items 12 and 19 instead of "risk of bias" (which is more applicable to systematic reviews of interventions) to include and acknowledge the various sources of evidence that may be used in a scoping review (e.g., quantitative and/or qualitative research, expert opinion, and policy document).

From: Tricco AC, Lillie E, Zarin W, O'Brien KK, Colquhoun H, Levac D, et al. PRISMA Extension for Scoping Reviews (PRISMAScR): Checklist and Explanation. Ann Intern Med. 2018;169:467–473. doi: 10.7326/M18-0850.

Online supplements are not copyedited prior to posting and the author(s) take full responsibility for the accuracy of all data.

e-Table 2: Full search strategy

Background<br>The search was informed by the PICO strategy where:<br>• P (problem or population): country-specific COPD guidelines<br>• I (intervention): not applicable<br>• C (comparison): high-income versus low and middle income countries and<br>• O (outcomes): characteristics, content, ethical, legal, social and economic aspects and adherence to the Institute of Medicine (IOM) standards.<br>To identify as many guidelines as possible, a sequential approach was taken. This started with PubMed/Embase searches, followed by targeted searches in specific databases and through reaching out through the GACD network.
PubMed search<br>The PubMed search was performed on February 15, 2019 at https://www.pubmed.gov.<br>The following search string was used:<br>(((("guideline"[All Fields] OR "consensus"[All Fields]) OR "protocols"[All Fields]) OR "standards"[All Fields]) OR "recommendations"[All Fields]) AND "COPD"[All Fields]<br>No filters were applied.<br>This search yielded 3,030 hits.
EMBASE search<br>The EMBASE search was performed on February 18, 2019 at www.embase.com.<br>The following search string was used:<br>('guideline'/exp OR 'guideline' OR 'consensus'/exp OR 'consensus' OR 'protocols' OR 'standards'/exp OR 'standards' OR 'recommendations'/exp OR 'recommendations') AND ('copd'/exp OR 'copd')<br>No filters were applied.<br>This search yielded 9,376 hits.
After screening the Pubmed and EMBASE search results, 133 hits remained. After removing duplicates and applying in- and exclusion criteria (see e-Figure 1), a total of 27 different national COPD guidelines were identified for which the most recent version was obtained. These were **Argentina, Australia/NZ, Brazil, Canada, Czech Republic, Denmark, Finland, France, Germany/Austria, India, Italy, Japan, Mexico, Netherlands, Poland, Russia, Saudi Arabia, Slovenia, Singapore, South Africa, South Korea, Spain, Switzerland, Tunisia, Turkey, UK** and **USA.**
GACD network<br>Suggested by GACD network members: 13 (**Bangladesh, Chile, Colombia, Greece, Ireland, Malaysia, Norway, Portugal, Romania, Slovak republic, Sweden, Taiwan, Vietnam**)<br>Identified after targeted outreach and online searches: 21 (**Belarus, Bulgaria, China, El Salvador, Guatemala, Hungary, Indonesia, Iran, Kazakhstan, Kyrgyz Republic, Moldova, North Macedonia, Peru, Philippines, Serbia, Qatar, Tajikistan, Thailand, Ukraine, United Arab Emirates, Uzbekistan**)
IPCRG database search<br>The International Primary Care Respiratory Group database was searched on June 5, 2019 for COPD guidelines at https://old.theipcrg.org/display/ResMapping/Overview+of+national+guidelines+used+by+primary+care<br>The IPCRG database listed 21 countries for which the status of a national COPD guideline was reported. These 21 were Australia, Bangladesh, Canada, Chile, Cyprus, Greece, India, Ireland, Italy, Netherlands, New Zealand, Norway, Pakistan, Poland, Portugal, Singapore, Spain, Sri Lanka, Sweden, UK and Vietnam. Of those, for most of all (N=17) except for Cyprus, Greece, Pakistan and Sri Lanka (N=4) the existence of a national COPD guideline was reported. For Cyprus, it was indicated that GOLD guidelines were used but that a national guideline was planned.<br>Following this database search, N=7 country guidelines (**Bangladesh, Chile, Ireland, Norway, Portugal, Sweden and Vietnam**) not found in PubMed/Embase (but suggested by GACD members) were identified.
Trip database<br>The Trip database was searched (https://www.tripdatabase.com/search?categoryid=4&criteria=copd) on June 28, 2020 and repeated on August 7, 2020 for COPD guidelines focusing on countries where no guidelines were yet identified. The most recent search by searching on "COPD" yielded 77,443 hits. Narrowing down to "Guidelines" yielded 2,390 hits of which 2055 related to Australia/New Zealand (162), USA (1,186), Canada (209), and the UK (499). A total of 335 related to other countries, yet the majority to societal guidelines (e.g. ERS) or no COPD specific guidelines. National COPD guidelines were identified for Singapore, but this one was already identified.
GIN database<br>The International Guidelines Library was searched on August 6, 2020 for COPD guidelines at https://g-i-n.net/library/international-guidelines-library/international-guidelines-library/@@guideline_search_results?basic-searchable-text=copd&type=basic&action=Search&advanced-authors=&diseases=&meshterm=&search=<br>This search yielded 19 guidelines from 8 countries (Australia, Belgium, Denmark, Germany, Finland, Netherlands, Spain and United States). Only for Belgium we had not previously find a guideline, yet the actually listed guidelines were only focusing on treatment of exacerbations or physical therapy and did therefore not fulfill our inclusion criteria.

Online supplements are not copyedited prior to posting and the author(s) take full responsibility for the accuracy of all data.

e-Table 3: Background IOM standards

Background<br>In 2008, through the *Medicare Improvements for Patients and Providers Act*, the American Congress, requested the Institute of Medicine (IOM) to undertake a study on the optimal methods for developing clinical practice guidelines. Following this request, in 2011, the Institute of Medicine (IOM) published *Clinical Practice Guidelines We Can Trust*. This book explores questions surrounding guideline quality and standards. Notably, it formulates eight standards for developing trustworthy clinical guidelines emphasizing: (1) establishment of transparency, (2) guideline development group composition, (3) management of conflict of interest, (4) systematic review—guideline development intersection, (5) rating strength of guideline recommendations, (6) articulation of recommendations, (7) external review & (8) updating.
Application of IOM standards to COPD guidelines<br>In order to establish whether IOM criteria were met, for each COPD guideline the IOM standards were applied as follows:<br>1. **Establishment of transparency**: *Was the funding of this guideline's development specified?* (yes/no)<br>2. **Guideline development group composition**: *What was the profession of the authors of this Guideline (multiple answers possible)?* (physician specialists, GPs, nurses, pharmacists, physiotherapists, dieticians, patients, other, unknown). If more than one discipline was ticked, this criterion was deemed met.<br>3. **Management of conflict of interest**: Were conflicts of interest or disclosures of the *authors of this Guideline openly declared*? (yes/no). If yes, this criterion was deemed met.<br>4. **Systematic review—guideline development intersection**: *Was this Guideline based on systematic literature reviews?* (yes/no/unclear [not stated]). If yes, this criterion was deemed met.<br>5. **Rating strength of guideline recommendations**: *For each treatment recommendation regarding benefit or harm, was a hierarchical grading (e.g. use of a systematic assessment such as the GRADE approach) of the confidence in the evidence for the recommendations provided (e.g. A. based on Meta-analyses of multiple RCTs, B. single RCT and C, expert opinion or case reports)?* (yes/no/unclear [not stated]). If yes, this criterion was deemed met.<br>6. **Articulation of recommendations**: *Are the Guideline recommendations actionable, that is, tailored to local COPD context (such as epidemiology, risk factors) and locally available evidence and resources? (contrasting to simply copying GOLD guidelines, which is often indirect evidence from other countries)* (yes/no). If yes, this criterion was deemed met.<br>7. **External review**: *Was this Guideline posted for external review and/or public comment?* (yes/no/unclear [not stated]). If yes, this criterion was deemed met.<br>8. **Updating**: *1. Year of current most recent guideline and 2. When is an update of this Guideline planned?* (around 2019/around 2020/later than 2020/unknown [not stated]). If unknown, this criterion was deemed not met.
Reference:<br>Institute of Medicine. Clinical practice guidelines we can trust [Internet], 2011. Available from : http://www.nap.edu/catalog/13058/clinicalpractice-guidelines-we-can-trust. Accessed 25 October 2019

Online supplements are not copyedited prior to posting and the author(s) take full responsibility for the accuracy of all data.

e-Table 4: Statistical comparisons

**Target audience**	**LMIC (N, %) (Total N=30)**	**HIC (N, %) (Total N=31)**	***p*-value (Pearson Chi-square; *<0.05)**
Specialists	27 (90%)	28 (90%)	0.966
GPs	28 (93%)	27 (87%)	0.414
Nurses	11 (37%)	24 (77%)	0.001*
Pharmacists	8 (27%)	14 (45%)	0.133
Physiotherapists	8 (27%)	17 (55%)	0.025*
Dieticians	3 (10%)	10 (32%)	0.034*
Patients	5 (17%)	4 (13%)	0.679
Others	9 (30%)	6 (19%)	0.334
Unknown	0 (0%)	2 (6%)	0.157
**Content**	**LMIC (N, %) (Total N=30)**	**HIC (N, %) (Total N=31)**	***p*-value (Pearson Chi-square; *<0.05)**
Epidemiology	18 (60%)	24 (77%)	0.142
Case finding	12 (40%)	26 (84%)	0.000*
Smoking cessation	28 (93%)	30 (97%)	0.534
Air pollution	14 (47%)	13 (42%)	0.710
Vaccination (both inf/pneu)	25 (83%)	29 (94%)	0.211
Exacerbations	29 (97%)	30 (97%)	0.981
Comorbidity	11 (37%)	24 (77%)	0.001*
Diet	14 (47%)	20 (65%)	0.161
Physical activity	26 (87%)	29 (94%)	0.367
Pharmacological treatment	30 (100%)	31 (100%)	-
Education	20 (67%)	21 (68%)	0.929
Alternative medicine	3 (10%)	1 (3%)	0.285
Vulnerable populations	2 (7%)	1 (3%)	0.534
**ELSE**	**LMIC (N, %) (Total N=30)**	**HIC (N, %) (Total N=31)**	***p*-value (Pearson Chi-square; *<0.05)**
Ethical	9 (30%)	9 (29%)	0.934
Legal	6 (20%)	11 (35%)	0.178
Social	8 (27%)	11 (35%)	0.457
Economic	8 (27%)	13 (42%)	0.210
**IOM item**	**LMIC (N, %) (Total N=30)**	**HIC (N, %) (Total N=31)**	***p*-value (Pearson Chi-square; *<0.05)**
Funding transparancy	6 (20%)	20 (65%)	0.000*
Multidisciplinary authors	16 (53%)	19 (61%)	0.530
Conflicts of interest reported	7 (23%)	23 (74%)	0.000*
Informed by systematic reviews	18 (60%)	22 (71%)	0.367
Strength of recommendations	16 (53%)	22 (71%)	0.155
Articulation of recommendations	15 (50%)	24 (77%)	0.026*
External review	16 (53%)	19 (61%)	0.530
Updates specified	7 (23%)	15 (48%)	0.042*
**IOM total**	**LMIC (mean, SD) (Total N=30)**	**HIC (mean, SD) (Total N=31)**	***p*-value (student's t-test; *<0.05)**
Mean number of IOM items fulfilled	3.37 (2.092)	5.29 (2.020)	0.001*

ELSE: Ethical, legal, social, economic; GP: general practitioner; HIC: high-income country; IOM: Institute of Medicine; LMIC: low and middle income country; SD: standard deviation

Online supplements are not copyedited prior to posting and the author(s) take full responsibility for the accuracy of all data.

e-Table 5: GNI per capita and population sizes 2018 for countries with COPD guideline

Country	GNI per capita in US dollars (2018), Atlas method Worldbank	Population (2018)
**HIC**		
Argentina	12,390	44,494,502
Australia/New Zealand	46,935*	29,877,869[#]
Austria/Germany	48,200*	91,774,959[#]
Canada	44,940	37,058,856
Chile	14,670	18,729,160
Czech republic	20,240	10,625,695
Denmark	60,140	5,797,446
Finland	48,280	5,518,050
France	41,080	66,987,244
Greece	19,770	10,727,668
Hungary	14,780	9,768,785
Ireland	61,390	4,853,506
Italy	33,730	60,431,283
Japan	41,310	126,529,100
Netherlands	51,260	17,231,017
Norway	80,610	5,314,336
Poland	14,100	37,978,548
Portugal	21,990	10,281,762
Qatar	61,150	2,781,677
Saudi Arabia	21,600	33,699,947
Singapore	58,770	5,638,676
Slovak Republic	18,260	5,447,011
Slovenia	24,580	2,067,372
Korea, Rep.	30,600	51,635,256
Spain	29,340	46,723,749
Sweden	55,490	10,183,175
Switzerland	84,410	8,516,543
Taiwan^	28,113	23,780,000
United Arab Emirates	40,880	9,630,959
United Kingdom	41,770	66,488,991
United States	63,080	327,167,434
TOTAL HICs with COPD guideline		1,187,740,576
TOTAL HICs in the world		1,210,312,147
No COPD guideline		22,571,571 (1.9%)
**LMIC**		
Belarus	5,670	9,485,386
Brazil	9,140	209,469,333
Bulgaria	8,860	7,024,216
China	9,460	1,392,730,000
Colombia	6,180	49,648,685
Guatemala	4,400	17,247,807
Iran, Islamic Rep.	5,470[&]	81,800,269
Kazakhstan	8,070	18,276,499
Malaysia	10,590	31,528,585
Mexico	9,180	126,190,788
Peru	6,470	31,989,256
North Macedonia	5,450	2,082,958
Romania	11,290	19,473,936
Russian Federation	10,230	144,478,050
Serbia	6,390	6,982,084
South Africa	5,750	57,779,622

Online supplements are not copyedited prior to posting and the author(s) take full responsibility for the accuracy of all data.

Thailand	6,610	69,428,524
Turkey	10,420	82,319,724
Bangladesh	1,750	161,356,039
El Salvador	3,820	6,420,744
India	2,020	1,352,617,328
Indonesia	3,840	267,663,435
Kyrgyz Republic	1,220	6,315,800
Moldova	2,980	3,545,883
Philippines	3,830	106,651,922
Tunisia	3,500	11,565,204
Ukraine	2,660	44,622,516
Uzbekistan	2,020	32,955,400
Vietnam	2,360	95,540,395
Tajikistan	1,010	9,100,837
TOTAL LMICs with COPD guideline		4,456,291,225
TOTAL LMICs in the world		6,383,958,209
No COPD guidelines		1,927,666,984 (30.2%)
**All countries with COPD guidelines**		
All countries with COPD guidelines	-	5,644,031,801
All countries in the world	-	7,594,270,356
No COPD guidelines	-	1,950,238,555 (25.7%)

GNI: gross national income; *mean GNI from two countries; #: sum of both countries; ^: no Worldbank data available, governmental data used instead; &: no 2018 Worldbank data available, 2017 used instead

Online supplements are not copyedited prior to posting and the author(s) take full responsibility for the accuracy of all data.

e-Table 6: Available COPD guidelines from low and lower-middle income countries

No	*Country*	*Year*	*Title*	*Author(s)*
1	Bangladesh	2016	National Guidelines Asthma & COPD	Asthma Association Bangladesh
2	El Salvador	2005	Guide of attention to Chronic Obstructive Pulmonary Diseases in primary health care	Chronic lung department from Ministry of Health
3	India	2013	Guidelines for Diagnosis and Management of Chronic Obstructive Pulmonary Disease	Jointly by Indian Chest Society (ICS) and the National College of Chest Physicians (NCCP)
4	Indonesia	2008	Diagnostic Guideline for COPD Treatment in Indonesia	Ministry of Health
5	Kyrgyz Republic	2015	Clinical guidelines for diagnosis and treatment of lung diseases at primary and secondary health care level	Sooronbaev T., Isakova G., Shabykeeva S. - pulmonologist
6	Moldova	2013	Chronic Obstructive Lung Disease - National Clinical Protocol	Moldova Ministry of Health
7	Philippines	2009	Clinical Practice Guidelines in the Diagnosis and Management of Chronic Obstructive. Pulmonary Disease (COPD) in the Philippines	Philippine College of Chest Physicians
8	Tunisia	2017	Chronic Obstructive Pulmonary Disease: Recommendations issued by Respiratory Diseases and Alergology Society of Tunisia	Samy Kammoun, Abdelaziz Hayouni, Hedia Ghrairi, et al
9	Ukraine	2013	Chronic Obstructive Pulmonary Disease. Adapted Evidence Based Clinical Guideline 2013	Multidisciplinary working party established by the Ministry of Health
10	Uzbekistan	2014	Clinical guidelines for diagnosis and treatment of lung diseases	Tashkent Institute of physician's re-training, Republic Special Scientific Medical center of Physiatry and Pulmonology
11	Vietnam	2018	Guidelines for diagnosis and treatment of COPD	Ministry of Health
12	Tajikistan*	2018	Guidelines for management of lung diseases at primary health care	Bandaev I.-MD, Akhmedova H., Babaeva L.

*low income country

Online supplements are not copyedited prior to posting and the author(s) take full responsibility for the accuracy of all data.

e-Table 7: Available COPD guidelines from upper-middle income countries

No	*Country*	*Year*	*Title*	*Author(s)*
1	Belarus	2012	Clinical guidelines for Chronic Obstructive Pulmonary Disease	Specialists from MoH of Belarus
2	Brazil	2013	COPD - Clinical Protocol and Therapeutic Guidelines	Ministry of Health
3	Bulgaria	2019	Pharmacotherapeutic guideline on pneumology and physiatry	Bulgarian Association of Lung Diseases
4	China	2018	Guideline for primary care of chronic obstructive pulmonary disease: practice version	Chinese Medical Association; Chinese Medical Journal Publishing House; Chinese Society of General Practice; Chronic Obstructive Pulmonary Disease Group of Chinese Thoracic Society; Editorial Board of Chinese Journal of General Practitioners of Chinese Medical Association; Expert Group of Guidelines for Respiratory System Disease
5	Colombia	2014	Evidence Based Clinical Practice Guideline for Prevention, Diagnosis, Treatment and Follow up of Adult Population with COPD	Colombian Ministry of Health, Colombian National Center for Investigation in Health Evidence and Technology
6	Guatemala	2015	Clinical Guideline based on Evidence 'Management of COPD'	Instituto Guatemalteco de Seguridad Social (IGSS)
7	Iran	2016	National Guidance for COPD	Ministry of Health COPD Committee
8	Kazakhstan	2013	Clinical guidelines for Chronic Obstructive Pulmonary Disease	Djanuzakov M., Seledtcov V.
9	Malaysia	2009	MANAGEMENT OF CHRONIC OBSTRUCTIVE PULMONARY DISEASE	MINISTRY OF HEALTH MALAYSIA
10	Mexico	2009	Diagnosis and Treatment of COPD - Clinical Guideline	Centro Nacional de Excelencia Tecnológica - Instituto Mexicano de Seguridad Social
11	Peru	2016	Peruvian Guideline of COPD	Peruvian Society of Pneumologists
12	Republic of North Macedonia	2013	Guideline for practicing Evidence-based medicine for Chronic Obstructive Pulmonary Diseases (COPD)	Ministry of Health
13	Romania	2017	Global Initiative for Chronic Obstructive Lung Disease Pocket Guide [Romanian translation]	GOLD Science Committee 2016
14	Russia	2016	Clinical guidelines for Chronic Obstructive Pulmonary Disease	Russian Respiratory Society
15	Serbia	2012	National guideline of good clinical practice for diagnosis and treatment of chronic obstructive pulmonary disease (COPD)	Working group for conduct of the national COPD guideline (led by prof Marija Mitić Milikić)

Online supplements are not copyedited prior to posting and the author(s) take full responsibility for the accuracy of all data.

16	South Africa	2011	Guideline for the management of chronic obstructive pulmonary disease - 2011 update	South African Thoracic Society
17	Thailand	2017	The guideline for patients with chronic obstructive pulmonary diseases B.E.2560	Thoracic Society of Thailand under Royal Patronage
18	Turkey	2017	CONSENSUS REPORT The View of the Turkish Thoracic Society on the Report of the GOLD 2017 Global Strategy for the Diagnosis, Management, and Prevention of COPD	Turkish Thoracic Society

e-Table 8: Available COPD guidelines from high income countries

No	*Country*	*Year*	*Title*	*Author(s)*
1	Argentina	2016	National Clinical Practice Guidelines for the Diagnosis and Treatment of COPD	Argentinian Ministry of Health
2	Australia/New Zealand	2019	COPD-X Plan: Australian and New Zealand Guidelines for the management of Chronic Obstructive Pulmonary Disease	Yang I et al for Lung Foundation Australia
3	Austria/Germany	2018	Guideline for the Diagnosis and Treatment of COPD Patients - Issued by the German Respiratory Society and the German Atemwegsliga in Cooperation with the Austrian Society of Pneumology	German Respiratory Society and Austrian Society of Pneumology
4	Canada	2007*	Canadian Thoracic Society recommendations for management of chronic obstructive pulmonary disease - 2007 update	Canadian Thoracic Society
5	Chile	2013	Ministry of Health Clinical Guideline for Ambulatory Treatment of Chronic Obstructive Pulmonary Disease	Chilean Ministry of Health
6	Czech republic	2013	Chronic Obstructive Pulmonary Disease: official diagnosis and treatment guidelines of the Czech Pneumological and Phthisiological Society; a novel phenotypic approach to COPD with patient-oriented care	The Czech Pneumological and Phthisiological Society; coordinator Vladimir Koblizek
7	Denmark	2017	Danish COPD guideline	Danish Society of Respiratory Medicine
8	Finland	2014	Diagnosis and Pharmacotherapy of stable chronic obstructive pulmonary airway disease: The Finnish Guidelines	Persons from various Finnish hospitals and universities
9	France	2009	Recommandation pour la pratique clinique: prise en charge de la BPCO. [Recommendation for the clinical practice management of COPD].	Societe de pneumologie de langue francaise

Online supplements are not copyedited prior to posting and the author(s) take full responsibility for the accuracy of all data.

10	Greece	2015	The development of 13 guidelines of general practice, to manage the most common diseases and health conditions in primary health care Code MIS: 464637. The COPD.	Tsiligianni Ioanna, Lintovoi Eleftheria, Chatzea Valeria, Christos Lionis. Clinic of Social and Family medicine, Faculty of Medicine, University of Crete
11	Hungary	2017	Medical guidelines for the diagnosis, treatment and management of chronic obstructive pulmonary disease (COPD)	Böszörményi Nagy, Gy. Balikó, Z. Somfay, A. Varga, J. - Pulmonology Section of Professional College, Hungary
12	Ireland	2009	Management of Chronic Obstructive Pulmonary Disease in General Practice	Owens et al for Irish Thoracic Society and ICGP (Irish College of General Practitioners)
13	Italy	2014	The clinical and integrated management of COPD	Working group appointed by the three major national respiratory societies (AIMAR, AIPO e SIMeR) and the Italian Society of General Medicine (SIMG).
14	Japan	2018	The JRS Guidelines for the Management of Chronic Obstructive Pulmonary Disease	The Japanese Respiratory Society
15	Netherlands	2015	NHG COPD Guideline	Dutch College of General Practitioners
16	Norway	2012	National guidelines for the diagnosis and monitoring of people with COPD	Directorate of Health
17	Poland	2014	Polish Respiratory Society Guidelines for Chronic Obstructive Pulmonary Disease	Paweł Śliwiński, Dorota Górecka, Ewa Jassem, Władysław Pierzchała,
18	Portugal	2019	Diagnosis and Treatment of Chronic Obstructive Pulmonary Disease in Adults.	Direção-Geral da Saúde (Directorate-General for Health): Prof Cristina Barbara
19	Qatar	2017	The assessment and management of chronic obstructive pulmonary disease in adults	Ministry of Public Health of Qatar (MOPH)
20	Saudi Arabia	2014	Saudi Guidelines for the Diagnosis and Management of COPD	The Saudi Initiative for Chronic Airways Diseases (SICAD) panel
21	Singapore	2017	Chronic Obstructive Pulmonary Disease: Ministry of Health Clinical Practice Guidelines 2/2017	Singapore Ministry of Health with a Work Group
22	Slovakia	2018	Chronic obstructive pulmonary disease. National guidelines for prevention and therapy 2018	Slovak pneumological and phthiseological society
23	Slovenia	2017	Recommendations for the management of patients with chronic obstructive pulmonary disease (COPD) at primary and specialist pulmonary levels in Slovenia	Pulmonology association of Slovenia
24	South Korea	2018	COPD Clinical Practice Guideline of the Korean Academy of Tuberculosis and Respiratory Disease	Korean Academy of Tuberculosis and Respiratory Disease

Online supplements are not copyedited prior to posting and the author(s) take full responsibility for the accuracy of all data.

25	Spain	2017	GUÍA DE PRÁCTICA CLÍNICA PARA EL DIAGNÓSTICO Y TRATAMIENTO DE PACIENTES CON ENFERMEDAD PULMONAR OBSTRUCTIVA CRÓNICA (EPOC) - GUÍA ESPAÑOLA DE LA EPOC (GesEPOC)	Working group of the 11 (eleven) medical societies than handle COPD in Spain, from primary care, internists, and respirologists, to nurses, physioterapists and critical care, among other
26	Sweden	2015	Treatment recommendations of COPD from the Swedish Medical Products Agency	Swedish Medical Products Agency was responsible. The guidelines are written by multiple professions
27	Switzerland	2018	Diagnosis, Prevention and Treatment of Stable COPD and Acute Exacerbations of COPD: the Swiss Recommendations 2018	Stolz et al
28	Taiwan	2017	Taiwan Guidelines for the Clinical Management of Chronic Obstructive Pulmonary Disease	Health Promotion Administration, Ministry of Health and Welfare; Cochrane Taiwan; Taiwan Society of Pulmonary and Critical Care Medicine; Taiwan Evidence-Based Medicine Association
29	United Arab Emirates/Abu Dhabi	2016	HAAD Guidelines for the Diagnosis and Management of Chronic Obstructive Pulmonary Disease (COPD)	Health Authority of Abu Dhabi
30	United Kingdom	2019	Chronic obstructive pulmonary disease in over 16s: diagnosis and management	UK National Institute for Health and Care Excellence (NICE)
31	USA	2011	Diagnosis and Management of Stable Chronic Obstructive Pulmonary Disease: A Clinical Practice Guideline Update from the American College of Physicians, American College of Chest Physicians, American Thoracic Society, and European Respiratory Society	ATS, ACCP, ACP, ERS

*Note that an update of the Canadian guidelines came out in 2019, just after data analysis and database lock of this work so it could not be included.

Online supplements are not copyedited prior to posting and the author(s) take full responsibility for the accuracy of all data.

e-Table 9: Stakeholders targeted by COPD guidelines

Country	*Specialists*	*General Practitioners*	*Nurses*	*Pharmacists*	*Physiotherapists*	*Dieticians*	*Patients*	*Others*
**LIC**								
Tajikistan	1	1	0	0	0	0	0	0
**LMIC**								
Bangladesh	1	1	1	1	1	0	0	0
El Salvador	0	1	0	0	0	0	0	0
India	1	1	0	0	0	0	0	0
Indonesia	1	1	1	1	1	1	1	1
Kyrgyz Republic	1	1	0	0	0	0	0	1
Moldova	1	1	1	0	0	0	0	1
Philippines	1	1	1	1	1	1	0	0
Tunisia	1	1	1	0	0	0	0	1
Ukraine	1	1	0	0	0	0	0	1
Uzbekistan	1	1	0	0	0	0	0	0
Vietnam	1	1	0	0	0	0	0	0
**UMIC**								
Belarus	1	1	0	0	0	0	0	0
Brazil	1	1	1	1	0	0	0	1
Bulgaria	1	1	0	1	0	0	0	1
China	0	1	0	0	0	0	0	0
Colombia	1	1	1	1	1	0	1	0
Guatemala	1	1	0	0	0	0	1	0
Iran	1	1	1	0	1	0	1	0
Kazakhstan	1	1	0	0	0	0	0	0
Malaysia	1	1	1	1	1	0	0	0
Mexico	1	1	0	0	0	1	1	0
Peru	0	0	0	0	0	0	0	0
Republic of North Macedonia	1	1	0	0	0	0	0	1
Romania	1	1	1	0	0	0	0	1
Russia	1	1	0	0	0	0	0	0
Serbia	1	1	0	0	0	0	0	0
South Africa	1	1	0	0	1	0	0	0
Thailand	1	1	1	1	1	0	0	0
Turkey	1	0	0	0	0	0	0	0
**HIC**								
Argentina	1	1	1	1	1	0	0	0
Australia/New Zealand	1	1	1	1	1	1	0	0
Austria/Germany	1	0	0	0	0	0	0	0
Canada	1	1	1	0	0	0	0	0
Chile	1	1	1	1	1	0	0	0
Czech republic	1	1	1	1	1	1	1	1
Denmark	1	1	1	1	1	1	0	0

Online supplements are not copyedited prior to posting and the author(s) take full responsibility for the accuracy of all data.

Finland	1	1	1	0	0	0	0	0
France	1	1	0	0	1	0	0	0
Greece	0	1	1	0	0	0	0	1
Hungary	1	1	0	0	1	1	0	1
Ireland	0	1	1	0	0	0	0	0
Italy	1	1	0	0	0	0	0	0
Japan	1	1	1	1	1	1	0	0
Netherlands	1	1	1	0	0	0	0	0
Norway	1	1	1	0	1	0	0	0
Poland	1	0	0	0	0	0	0	0
Portugal	1	1	1	0	0	0	0	0
Qatar	1	1	1	1	1	1	0	0
Saudi Arabia	1	1	1	1	1	1	0	0
Singapore	1	1	1	1	1	0	0	0
Slovakia	1	0	0	0	0	0	0	0
Slovenia	1	1	1	0	0	0	0	0
South Korea	1	1	1	0	0	0	1	0
Spain	1	1	1	1	1	0	1	1
Sweden	1	1	0	0	1	1	0	0
Switzerland*	0	0	0	0	0	0	0	0
Taiwan	1	1	1	1	1	1	0	0
United Arab Emirates/Abu Dhabi	1	1	1	1	1	1	0	0
United Kingdom	1	1	1	1	1	1	1	1
USA	1	1	1	1	0	0	0	1

*target audience not explicitly stated; 1=yes, 0= no

Online supplements are not copyedited prior to posting and the author(s) take full responsibility for the accuracy of all data.

e-Table 10: COPD care addressed by guidelines (LIC, LMIC)

Country	Epidemiology	Case finding	Smoking cessation	Pollution	Diagnosis	Vaccination^	Exacerbations	Comorbidities	Diet	Physical activity	Pharmacological	Education	Alternative medicine	Vlunerable populations
**LIC**														
Tajikistan	0	0	1	0	1	1	1	0	0	1	1	1	0	0
**LMIC**														
Bangladesh	1	0	1	0	1	1	1	0	1	1	1	1	0	0
El Salvador	1	0	1	0	1	0	1	0	0	0	1	0	0	0
India	1	0	1	1	1	1	1	1	1	1	1	1	0	0
Indonesia	1	1	1	1	1	0	1	1	1	1	1	1	1	1
Kyrgyzstan	0	1	1	1	1	1	1	0	0	1	1	1	0	0
Moldova	1	1	1	1	1	1	1	0	0	1	1	1	0	0
Philippines	1	1	1	1	1	1	1	1	1	1	1	0	0	0
Tunisia	1	1	1	0	1	1	1	0	0	1	1	1	0	0
Ukraine	0	1	1	0	1	1	1	1	1	1	1	1	1	0
Uzbekistan	0	0	1	1	1	0	1	0	0	1	1	1	0	0
Vietnam	1	1	1	1	1	1	1	1	0	1	1	0	0	0

^: both influenza/pneumococcal; 1=yes, 0= no

e-Table 11: COPD care addressed by guidelines (UMIC)

Country	Epidemiology	Case finding	Smoking cessation	Pollution	Diagnosis	Vaccination^	Exacerbations	Comorbidities	Diet	Physical activity	Pharmacological	Education	Alternative medicine	Vulnerable populations
Belarus	0	0	0	0	1	0	1	0	0	0	1	0	0	0
Brazil	1	1	1	0	1	1	0	1	1	1	1	0	0	0
Bulgaria	1	0	1	1	1	1	1	0	0	1	1	0	0	0
China	0	0	1	1	1	1	1	1	0	1	1	1	0	0
Colombia	1	1	1	1	1	1	1	0	1	1	1	1	0	0
Guatemala	1	0	1	0	1	1	1	1	1	0	1	1	0	0
Iran	0	0	1	1	1	1	1	0	0	1	1	1	0	0
Kazakhstan	0	0	1	0	1	1	1	0	0	1	1	1	0	0
Malaysia	1	0	1	0	1	1	1	0	1	1	1	1	0	0
Mexico	0	1	1	0	1	1	1	0	0	0	1	0	0	0
Peru	0	1	0	0	1	0	1	0	1	1	1	0	0	0
Republic of North Macedonia	0	0	1	0	1	1	1	0	0	1	1	0	0	0
Romania	0	1	1	1	1	1	1	1	1	1	1	1	0	0
Russia	1	0	1	0	1	1	1	0	0	1	1	1	0	0
Serbia	1	0	1	0	1	1	1	1	1	1	1	1	1	1
South Africa	1	0	1	1	1	1	1	0	0	1	1	0	0	0
Thailand	1	0	1	0	1	1	1	0	1	1	1	1	0	0
Turkey	1	0	1	1	1	1	1	1	1	1	1	1	0	0

1: both influenza/pneumococcal; 1=yes, 0= no

Online supplements are not copyedited prior to posting and the author(s) take full responsibility for the accuracy of all data.

e-Table 12: COPD care addressed by guidelines (HIC)

Country	Epidemiology	Case finding	Smoking cessation	Pollution	Diagnosis	Vaccination^	Exacerbations	Comorbidities	Diet	Physical activity	Pharmacologic al	Education	Alternative medicine	Vulnerable populations
Argentina	0	1	1	0	1	1	1	1	1	1	1	0	0	0
Australia/New Zealand	1	1	1	1	1	1	1	1	1	1	1	1	1	0
Austria/Germany	1	1	1	0	1	1	1	1	1	1	1	0	0	0
Canada	1	1	1	0	1	1	1	1	0	1	1	1	0	0
Chile	1	1	1	0	1	1	1	0	1	1	1	0	0	0
Czech republic	1	0	1	1	1	1	0	1	1	1	1	1	0	0
Denmark	1	1	1	1	1	1	1	1	1	0	1	0	0	0
Finland	1	1	1	0	1	1	1	0	0	1	1	0	0	0
France	1	0	1	0	1	1	1	1	1	1	1	0	0	0
Greece	1	1	1	0	1	1	1	1	1	1	1	1	1	1
Hungary	0	1	1	1	1	1	1	0	1	1	1	0	0	0
Ireland	1	1	1	1	1	1	1	1	0	1	1	1	0	0
Italy	0	1	1	0	1	1	1	1	1	1	1	1	0	0
Japan	1	0	1	0	1	1	1	1	1	1	1	1	0	0
Netherlands	1	1	1	1	1	0	1	1	1	1	1	1	0	0
Norway	1	1	1	0	1	1	1	1	1	1	1	1	0	0
Poland	1	1	1	1	1	1	1	1	0	1	1	0	0	0
Portugal	1	1	1	1	1	1	1	0	0	1	1	1	0	0
Qatar	0	1	1	0	1	1	1	0	0	1	1	1	0	0
Saudi Arabia	1	1	1	0	1	1	1	1	1	1	1	1	0	0
Singapore	1	1	1	0	1	1	1	1	0	1	1	1	0	0
Slovakia	1	0	1	1	1	1	1	1	0	1	1	1	0	0
Slovenia	1	1	0	0	1	1	1	0	1	1	1	1	0	0
South Korea	1	1	1	1	1	1	1	1	1	1	1	1	0	0
Spain	1	1	1	1	1	1	1	1	0	1	1	1	0	0
Sweden	1	0	1	0	1	1	1	1	1	1	1	0	0	0
Switzerland	1	1	1	0	1	1	1	1	0	1	1	0	0	0
Taiwan	1	1	1	1	1	1	1	1	1	1	1	1	0	0
United Arab Emirates/Abu Dhabi	0	1	1	1	1	1	1	1	1	1	1	1	0	0
United Kingdom	0	1	1	0	1	1	1	1	1	1	1	1	0	0
USA	1	1	1	0	1	0	1	0	0	0	1	1	0	0

^: both influenza/pneumococcal; 1=yes, 0= no

Online supplements are not copyedited prior to posting and the author(s) take full responsibility for the accuracy of all data.

e-Table 13: Ethical, Legal, and Socio-economic (ELSE) Considerations (LIC, LMIC) and dissemination

Country	Ethical considerations	Legal considerations	Social considerations	Economic considerations	Dissemination plan
**LIC**					
Tajikistan	1	0	1	0	1
**LMIC**					
Bangladesh	0	0	0	0	3
El Salvador	0	0	0	0	2
India	0	0	0	1	2
Indonesia	1	1	1	1	2
Kyrgyzstan	1	0	1	0	1
Moldova	0	0	0	0	1
Philippines	1	0	0	0	1
Tunisia	0	0	0	0	2
Ukraine	1	1	1	0	3
Uzbekistan	0	0	0	0	3
Vietnam	0	1	0	0	2

Column dissemination plan: 1= yes; 2= no; 3= unknown (not stated); Other columns: 1=yes, 0=no

e-Table 14: Ethical, Legal, and Socio-economic (ELSE) Considerations (UMIC) and dissemination

Country	Ethical considerations	Legal considerations	Social considerations	Economic considerations	Dissemination plan
Belarus	0	0	0	0	3
Brazil	0	0	0	0	2
Bulgaria	0	0	0	1	3
China	0	0	0	0	3
Colombia	0	0	1	1	1
Guatemala	0	1	0	0	2
Iran	0	0	0	0	3
Kazakhstan	0	0	0	0	3
Malaysia	0	0	0	1	3
Mexico	0	0	0	0	2
Peru	0	1	0	1	1
Republic of North Macedonia	1	0	0	0	1
Romania	1	0	0	0	2
Russia	1	0	1	0	3
Serbia	0	0	1	0	2
South Africa	0	0	0	0	0
Thailand	1	1	0	1	3
Turkey	0	0	1	1	2

Column dissemination plan: 1= yes; 2= no; 3= unknown (not stated); Other columns: 1=yes, 0=no

Online supplements are not copyedited prior to posting and the author(s) take full responsibility for the accuracy of all data.

e-Table 15: Ethical, Legal, and Socio-economic (ELSE) Considerations (HIC) and dissemination

Country	Ethical considerations	Legal considerations	Social considerations	Economic considerations	Dissemination plan
Argentina	0	0	0	1	3
Australia/New Zealand	1	0	1	1	3
Austria/Germany	1	1	0	1	2
Canada	0	1	1	0	3
Chile	0	0	0	0	1
Czech republic	0	1	1	1	1
Denmark	1	1	1	0	2
Finland	0	0	0	0	3
France	0	0	0	0	2
Greece	0	0	1	0	1
Hungary	0	0	0	1	3
Ireland	0	0	0	0	3
Italy	0	1	1	1	2
Japan	1	1	0	1	3
Netherlands	1	1	1	1	1
Norway	1	0	0	1	2
Poland	0	0	0	0	2
Portugal	0	0	1	0	1
Qatar	0	0	0	0	3
Saudi Arabia	1	0	0	0	3
Singapore	0	0	1	1	3
Slovakia	0	1	0	0	2
Slovenia	0	0	0	0	2
South Korea	0	0	0	0	1
Spain	0	0	1	1	1
Sweden	0	1	0	1	2
Switzerland	0	0	0	0	3
Taiwan	1	1	0	0	1
United Arab Emirates/Abu Dhabi	0	0	0	0	3
United Kingdom	1	1	1	1	1
USA	0	0	0	0	1

Column dissemination plan: 1= yes; 2= no; 3= unknown (not stated); Other columns: 1=yes, 0=no

Online supplements are not copyedited prior to posting and the author(s) take full responsibility for the accuracy of all data.

e-Table 16: IOM Standards fulfilled (LIC, LMIC)

Country	Transparency	COI	Multidisciplinary approach	Systematic review	Strength of recommendations	Articulation	External review	Updates
**LIC**								
Tajikistan	0	0	1	0	1	1	0	0
**LMIC**								
Bangladesh	0	1	1	0	0	1	0	1
El Salvador	0	0	1	0	0	0	0	0
India	0	0	1	0	1	0	1	0
Indonesia	0	0	0	1	1	1	1	0
Kyrgyzstan	0	0	1	1	1	1	1	1
Moldova	0	0	1	1	0	0	1	0
Philippines	0	0	1	1	1	0	0	0
Tunisia	0	0	1	1	0	0	1	0
Ukraine	0	0	1	1	1	1	1	0
Uzbekistan	0	0	0	0	0	0	0	0
Vietnam	0	0	1	0	0	0	0	0

1=yes, 0=no

e-Table 17: IOM Standards fulfilled (UMIC)

Country	Transparency	COI	Multidisciplinary approach	Systematic review	Strength of recommendations	Articulation	External review	Updates
Belarus	0	0	0	0	0	1	0	0
Brazil	0	1	1	1	0	1	1	0
Bulgaria	0	0	0	0	0	1	1	0
China	0	0	1	0	0	1	0	0
Colombia	1	1	1	1	1	1	1	1
Guatemala	0	1	1	1	1	0	0	0
Iran	0	0	0	1	0	1	0	0
Kazakhstan	0	0	0	0	0	0	0	0
Malaysia	1	1	1	1	1	1	1	0
Mexico	1	0	1	1	1	0	1	0
Peru	0	0	0	1	1	0	1	1
Republic of North Macedonia	0	0	0	1	0	0	0	1
Romania	1	1	0	1	1	0	1	0
Russia	0	0	0	0	1	1	1	1
Serbia	0	0	0	0	0	0	0	0
South Africa	0	0	0	1	1	0	1	0
Thailand	1	0	0	1	1	1	0	0
Turkey	1	1	0	1	1	1	1	1

1=yes, 0=no

Online supplements are not copyedited prior to posting and the author(s) take full responsibility for the accuracy of all data.

e-Table 18: IOM Standards fulfilled (HIC)

Country	Transparency	COI	Multidisciplinary approach	Systematic review	Strength of recommendations	Articulation	External review	Updates
Argentina	1	1	1	1	1	0	0	0
Australia/New Zealand	1	1	1	1	1	1	1	1
Austria/ Germany	1	1	0	1	1	1	1	0
Canada	1	1	0	1	1	1	1	0
Chile	1	1	1	1	1	1	0	0
Czech republic	1	1	1	1	0	1	1	1
Denmark	0	1	1	0	0	1	0	1
Finland	0	0	0	1	1	0	0	0
France	1	1	1	1	0	0	1	0
Greece	1	1	1	1	1	1	1	1
Hungary	1	1	0	1	1	1	1	1
Ireland	0	0	0	1	1	1	0	0
Italy	0	1	1	0	0	0	0	1
Japan	0	1	0	0	1	1	1	0
Netherlands	1	1	1	1	1	1	1	1
Norway	1	1	1	0	0	1	1	1
Poland	0	0	0	1	0	1	1	0
Portugal	1	1	0	0	1	0	1	1
Qatar	1	1	1	1	0	1	0	1
Saudi Arabia	1	1	1	1	1	1	1	0
Singapore	0	0	1	0	1	1	0	1
Slovakia	0	0	0	0	0	1	1	1
Slovenia	0	0	1	0	1	1	1	0
South Korea	1	1	1	1	1	1	1	1
Spain	1	1	1	1	1	1	0	1
Sweden	1	1	1	1	1	1	0	0
Switzerland	1	0	0	1	1	0	0	0
Taiwan	0	1	0	1	1	1	1	1
United Arab Emirates/Abu Dhabi	0	0	0	0	0	0	0	0
United Kingdom	1	1	1	1	1	1	1	0
USA	1	1	1	1	1	1	1	0

1=yes, 0=no

Online supplements are not copyedited prior to posting and the author(s) take full responsibility for the accuracy of all data.
